# Southern Surgical and Gynæcological Association

**Published:** 1896-01

**Authors:** 


### Society Reports.

---

### SOUTHERN SURGICAL AND GYNÆCOLOGICAL ASSOCIATION.

### Abstract of Proceedings of the Eighth Annual Meeting, Washington, D. C., November 12, 13 and 14, 1895.

FIRST DAY.—MORNING SESSION.

The Association met in the banquet hall of the Hotel Shoreham at 10 A. M., and was called to order by the president, Dr. L. McLane Tiffany, of Baltimore, Md.

DR. H. C. BUSEY, of Washington, delivered an address of welcome on behalf of the medical profession of the District of Columbia. In his closing remarks, Dr. Busey said: "I solicit your aid and co-operation in our effort to secure the protection of our people from the horde of imposters and charlatans, which you have driven from your borders by the enactment and enforcement of medical practice laws, and which has made the District of Columbia a common rendezvous where the most atrocious methods of the charlatan and mercenary impositions are openly and flagrantly committed, to the wrong, injury and robbery of its citizens. You represent the most influential and intelligent class of suffragists, for whose aid on the hustings and at the polls we plead.

"To state the deplorable condition of this District fully and broadly, there are five medical schools and several medical societies chartered by acts of congress, or under the general incorporation law authorized and empowered to license persons to practice the art and science of medicine, without any uniformity, and by some, without any standard of qualification beyond the ability and willingness of the applicant to pay the required fees, or give promissory notes for such payment; and under the provisions of the general incorporation law, any dozen of persons can obtain a charter upon payment of the fee for recording the same, authorizing them as a body corporate to confer the degree of M. D. at their pleasure and will. Such is the status of this federal territory, which is under the exclusive jurisdiction of the highest tribunal of legislation in the land, made up of the representatives and senators from forty-nine states and territories, which have enacted medical practice laws for the protection and welfare of their citizens. Take these facts home with you and re-echo them throughout the length and breadth of the land, that such criminal neglect, not less disgraceful and scandalous than the slums of vice, may not continue to afflict the citizens of the federal territory."

PRESIDENT TIFFANY then responded to the address of welcome for the Association.

After some announcements had been made by Dr. Joseph Taber Johnson, of Washington, chairman of the committee of arrangements, the reading of papers was taken up.

DR. BEDFORD BROWN, of Alexandria, Va., read a paper entitled

### PERSONAL EXPERIENCE IN THE TREATMENT OF STAB WOUNDS OF THE INTESTINES AND PERITONEUM.

At the outset, the author stated that about 130 cases of stab wounds of the peritoneum and intestines had come under his care during his entire professional experience in both private and military practice. In less than one-third of the cases, the intestines were wounded. "It is a little remarkable," said the author, "that there should be such a disproportion in the number of intestinal wounds in these cases; in other words, it is a singular fact that in a large majority of abdominal wounds the intestines escape injury, even when such wounds are extensive."

Transverse and longitudinal stab wounds of the intestines were then considered at length. Dr. Brown regards the sabre wound as one of the most dangerous, in its immediate and remote results. If the edge and point of the sabre are sharp, the wound inflicted is large and deep. The weapon cutting through the intestines and mesentery, usually passing through the abdomen, severs large blood vessels and causes frightful hæmorrhage, which is speedily fatal. He had only seen three sabre wounds of the abdomen, and they ended fatally in a short time. The stiletto is a dangerous instrument, as it almost invariably enters an intestine or other organ. It does not kill by hæmorrhage, usually, but makes an opening in the intestines sufficiently large to permit the escape of a small quantity of fæcal matter, causing septic inflammation. It is one of the most difficult of all intestinal wounds to detect.

The diagnosis of intestinal wounds was then dwelt upon, reference being made to Senn's hydrogen gas test to detect wounds of the intestine. While he considered it a useful test, in remote sections of the country, far from large cities and towns, it is not practicable, because of the impossibility of procuring the apparatus and generating the gas. In all abdominal stab wounds, the author's rule has been, after cleansing the hands and thoroughly disinfecting them, to insert the index finger and explore the intestine to ascertain if there is an opening. In certain cases a wound may exist in the intestine, but it may be so small as to escape detection. But if the intestine is wounded, whether we can insert the finger or not, there is always more or less extravasation of fæcal matter and gases, and if the finger comes in contact with this matter, it is certain to retain for some length of time the peculiar odor of human fæces. This will always afford positive evidence of an intestinal wound.

In treating simple wounds of the peritoneum it has been the author's rule to close them with silver wire sutures after thorough disinfection. Formerly, these wounds were closed, regardless of antiseptic measures, except that the wound was washed with hot water and soap. On the battlefield and in field hospitals, wounds were washed with any water that was convenient, and were not washed at all when water could not be obtained. Previous to the introduction of antiseptic treatment in dressing wounds, but little attention was paid to the condition of the instruments, sutures, sponges, or dressings, except the practice of ordinary cleanliness, and the percentage of cases healing by first intention of simple wounds of the peritoneum was large. In dressing simple wounds of the peritoneum, scrupulous attention should be paid to the laws of cleanliness. In treating wounds of the intestines, two vital procedures are necessary: (1) Complete and thorough closure of he intestinal wound, and (2) to cleanse the peritoneal cavity of all fæcal matter, blood and gases escaping from the intestine.

Dr. Brown then described a simple method of reducing a protruded intestine in stab wounds. He takes two long, slender, curved needles, threaded each with a silken cord 10 or 12 inches long. One of these needles is passed midway through the margin of the wound, the other needle is passed through the opposite margin, and then each cord is tied in a separate loop. These cords are drawn in opposite directions by two assistants, upwards and outwards, firmly and tightly. By this means, the wound is made to expand or gape widely, and at the same time the walls of the abdomen, for a large area around the wound, are considerably elevated above the intestines, while the patient reclines in the dorsal position, and a considerable vacuum is in this way created and the intestines will glide back without force or manipulation to fill this newly created vacuum.

DR. RICHARD DOUGLAS, of Nashville, said, that in peritoneal wounds, we always have a mixed infection, which is more serious than an infection from the colon bacillus. Peritonitis, whether local, adhesive, general, or septic, should be considered of bacterial origin. In closing the abdominal wound, the peritoneum should always be approximated, as by so doing we lessen the danger of hernia.

DR. C. A. L. REED, of Cincinnati, expressed himself as being apprehensive about mere exploration with the finger to detect stab wounds of the intestines. However erudite the tactile sense of the surgeon may be, at times it proves misleading, and therefore, in certain cases, it is exceedingly important to enlarge the original wound, and that part of the viscera lying immediately beneath it should be brought out and carefully inspected. He believes with Dr. Douglas that the peritoneal margins should be carefully approximated.

DR. JAMES EVANS, of Florence, S. C., related an instance where nine men had received chest wounds by bayonets during the war, the bayonet having been previously stuck in the ground, and yet all the men recovered. He attributes their recovery to the form of wound made by the bayonet. In another case, a man had been shot within half an inch of the navel. He had no rise of temperature, yet when he saw the patient the omentum had extruded to the size of both his hands. He applied a double ligature, then put a piece of adhesive plaster over the surface, and the man recovered. He had frequently seen gunshot wounds of the abdomen, during the war, in which there was extravasation of fæcal matter through the wound, but unaccompanied by shock.

DR. A. VANDER VEER, of Albany, had made it a practice to first inquire carefully as to the kind of weapon with which the wound is made. He had seen several wounds inflicted by bayonets during the war, but does not remember of having seen the intestines or stomach penetrated by them. There should be no delay in treating stab wounds. The surgeon should act promptly and not wait for symptoms to present themselves. Just as a case of perforative appendicitis will terminate fatally in a short time, so will stab wound of the intestinal tract, unless timely interference is resorted to.

DR. HUGH T. NELSON, of Charlottesville, Va., said the necessity of enlarging the abdominal wound, under all circumstances was an imperative one. Four years ago he saw a case in which the small bowel was wounded by a knife, and the patient refused operation for twenty-four hours, believing that this viscus was not cut. Symptoms became alarming, and the patient finally consented to have an operation performed. Dr. Nelson opened the abdomen by a long incision, finding it impossible to remove from the peritoneal cavity the extruded contents of the bowels, owing to the fact that an adhesive inflammation had taken place and had agglutinated them to the bowel so firmly that he could not wash them away. He attempted to resect the peritoneum into the pelvic cavity where the fæcal matter had burrowed, but could not do so. Peritonitis became general and the patient died. The sooner the abdominal incision is enlarged in stab wounds, the better.

DR. GEORGE ROSS, of Richmond, Va., asked whether there was any way to distinguish between the symptoms of nervous shock and shock due to hæmorrhage.

DR. BROWN replied that one of the most unerring symptoms was rapid reduction of temperature, but there was no symptom that would enable the practitioner to distinguish accurately between the different forms of shock except the gravity of the condition.

DR. W. E. B. DAVIS, of Birmingham, desired to speak of the point in reference to injuries of the gall bladder. The essayist referred to the fact that injury to this viscus would produce a septic peritonitis. He thought that an injury that would produce peritonitis would speedily result in death, if there was a large escape of bile into the peritoneal cavity, but he does not believe it is a septic peritonitis; that in the majority of cases the shock following abdominal injuries is due to hæmorrhage; and that hemorrhage plays a more important role in the production of symptoms in intestinal injuries than we were formerly led to believe; in fact, it was hæmorrhage from these wounds that frequently caused death.

DR. JOHN D. S. DAVIS, of Birmingham, expressed himself, in regard to the diagnosis of intestinal wounds, as having very little confidence either in Senn's hydrogen gas test, or the flushing method spoken of by the essayist. He had seen perforative wounds of the abdominal viscera where it was impossible, from their character and location, to flush the abdominal cavity through the opening sufficiently to thoroughly cleanse it. In addition to the three forms of shock mentioned by the essayist, there should be added the shock of sepsis.

DR. BROWN, in closing, agreed with Dr. W. E. B. Davis that all cases of violent or dangerous shock were due to hæmorrhages. In regard to approximating the peritoneum, he had always left it untouched in closing small wounds in the abdominal wall, and had found it good practice.

### REPORT OF SEVEN CASES OF ABDOMINAL SURGERY IN WHICH THE MURPHY BUTTON WAS APPLIED.

DR. A. VANDER VEER, of Albany, N. Y., read a paper so entitled. The author stated that the seven cases he desired to present had a bearing upon the use of the Murphy button, which is now receiving attention both in this country and abroad, and as a method of intestinal anastomosis is being placed thoroughly on its merits. It is difficult to understand some of the unfavorable reports made by English and German surgeons, when we contrast the very successful results indicated by so many of our American operators in the practical application of this mechanical device. Perhaps there is no part of surgery that within the past quarter of a century has presented so much in theory, and in which there has been so much disappointment when practical use has been made of the suggestions, as in the field of abdominal work, with all its complications. In other words, how much we have changed, from time to time, our methods of treatment of many complications; and yet, withal, there have come certain reliable advances that have met all requirements for which they were indicated, leaving permanently in our possession the comforting thought that a grand progress, in the sum total, has been made; that we can treat all manner of pathological conditions, traumatisms, malformations, etc., of the intestinal tract and abdominal cavity with less embarrassment than, perhaps, in either part of the body, and yet there are very few portions of the human system upon which we operate where more rapid thought and better judgment are to be employed than in abdominal work.

CASE I.—The first case that Dr. Vander Veer reported was one in which gastrointestinal anastomosis was made for carcinoma of the pyloric end of the stomach by means of the medium-sized Murphy button, between the upper end of the jejunum and great curvature of the stomach. Patient was comfortable after the operation, but died from exhaustion on the third day.

CASE II.—Carcinoma of the sigmoid flexure, removal and end-to-end anastomosis. Operation consisted of removing a mass in connection with the sigmoid flexure three inches in length, and an anastomosis of the large intestine by means of the button. Cause of constriction was found to be carcinoma. Patient died from exhaustion on the eleventh day, but was much exhausted and emaciated previous to operation.

CASE III.—Removal of gallstones from the gall bladder using the long drainage tube button. Recovery.

CASE IV.—Removal of eight inches of the small intestine with papillomatous ovarian cyst. End-to-end anastomosis by the button. Recovery.

CASE V.—Anastomosis of the gall bladder with small intestine. Recovery.

CASE VI.—Operation revealed a tumor the size of a cocoanut in the immediate vicinity of the umbilicus, a portion the size of a silver dollar implicating the umbilicus and in a gangrenous condition. On making incision there was found a strangulated hernia and many old, firm adhesions. Peritoneum intensely congested; very dark color. Loop of small intestine included in tumor and gangrenous for space of ten inches. Vessels in mesentery secured and this portion of the intestine excised. Murphy button used for end-to-end anastomosis. Button passed thirteen days after operation, followed by a large movement of the bowels. Uninterrupted recovery.

CASE VII.—A diagnosis of biliary calculi was made in this case. The usual incision was made for exploration of the gall bladder, and the gall bladder was found to contain about two ounces of bile, and through the walls and down into the cystic duct could be felt a number of small calculi. There were some adhesions. The author made use of the long drainage tube button to the fundus of the bladder and closed the wound, after a careful examination for any possible cancerous mass, which was not found to be the case, then placed the patient in bed. He regarded the use of the button in this case as a saving of time, leaving the patient in good condition for removal of the gallstones later. Several days after he had made the exploratory incision, the attending physician removed five irregularly shaped calculi, which Dr. Vander Veer exhibited. At this time, the patient began to show marked symptoms of cerebral anæmia, with delirium, which continued, patient finally passing into a comatose state and dying.

Dr. Vander Veer said that although the cases he had reported were not many, yet they covered a field in which the Murphy button might be made use of so readily and easily, and the results so satisfactory, that he had considered them worthy of attention as having a bearing upon statistics. The Murphy button will not answer for every lesion about the intestinal tract, but surely has its sphere of usefulness, in that it is clean, easily handled, and saves the patient from a much longer operation, when time alone is the great desideratum, which cannot be secured by some of the other methods.

DR. H. GRANT, of Louisville, followed with a paper entitled

### INTESTINAL RESECTION AND ANASTOMOSIS.

He said there had always been a division of judgment upon the question of immediate suture in acute obstruction or injury requiring resection of the intestine, which even the improvements in technique and means of aid in operative work have not adjusted. The members were all familiar with the Murphy button, and, doubtless, many had employed it. What it is intended to do, it does well; but too often it does what is not intended, and disaster and death result. There is abundant evidence that it becomes a foreign body; that it occasions spreading necrosis, which involves the peritoneal coat; that reconstruction takes place after lateral anastomosis; that fatal results are frequently directly attributable to its use, besides other less important objections.

Lateral anastomosis is now beyond all question the most acceptable method of resection of the continuity of the bowel, if we exclude the button. It is best accomplished by direct suture, and direct suture is difficult to execute except in very skilled hands. In order to facilitate direct suture, Dr. Grant presented a device for clamping opposing surfaces of the bowel, cutting off fenestra between them, and retaining them so opposed until the suture can be completed. He then demonstrated the *modus operandi* of this device.

He had experimentally used the clamp sixteen times with fourteen consecutive recoveries, but had had but one opportunity to use it in practice. May 25, he operated on Mrs. E., aged 53 years, who had a fæcal fistula at the right femoral opening, the result of a strangulated hernia, operated on eight months ago. An incision was made just above Poupart's ligament near the fistulous opening. The fingers easily liberated the intestine, which presented an opening occupying half its lateral surface and as large as a quarter of a dollar. The mesentery was greatly thickened; the distal segment of the bowel was reduced in size, the proximal dilated at the site of the fistula. About four inches of the intestine was resected; the blades of the clamp were applied opposite the mesenteric borders of each segment, and the anastomosis made as above-described. After suturing, the communication between the opposing surfaces was found ample. The cut ends were then invaginated and the anastomosis returned; the abdominal wall closed with silkworm gut sutures; the site of the fistula curetted and filled with iodoform gauze and the patient put back to bed in forty-two minutes. There was very little shock. At the present time, the patient is well.

The advantages of this method over the other aids, except the button, are manifest. Not only does it do away with the foreign body, but it cuts an opening three or four inches long at the fenestra. It is fully as easily accomplished, and takes less time. It is no more difficult to use than is the button, but the operation cannot be so quickly completed, as the invagination of the ends is not necessary after the end-to-end approximation by the button. The clamp merely makes direct suture easy to an ordinarily skilled hand.

DR. C. A. L. REED said experience would establish the fact that the Murphy button ought not to be used in approximation of the large intestine, for the reason that the intestinal contents were not sufficiently liquid to pass through the small opening in the button. In the small intestine it is different. There we have liquid contents that will pass through the opening in the button and the approximation is satisfactorily accomplished. Dr. Reed reported a case of resection of the sigmoid for malignant disease (which terminated fatally) in substantiation of the above remarks, the anastomosis being made by means of the Murphy button. He commended the device presented by Dr. Grant, and although he preferred the end-to-end procedure, he would try the device in the next case in which he performed lateral anastomosis. Cholecystenterostomy by means of the Murphy button was one of the easiest, neatest and altogether most satisfactory operations to surgery.

DR. JOSEPH M. MATHEWS, of Louisville, said he had taken occasion more than once to call attention to the difficulty that attends diagnosticating tumor of any kind in the sigmoid flexure. Time and again he had been mistaken, as he believed others had in supposing that he had malignant tumor of the sigmoid when he had not, and supposing, on the other hand, that he did not have, when he really did. A few years ago a patient was brought to him from an adjoining state, and from evidence outside of palpation, he believed that the man had malignant trouble of the sigmoid flexure. A few days thereafter he was taken to Chicago, and examined by a very eminent surgeon, who positively stated that there was no tumor of any kind in the flexure, and advised the patient to go home and go to work. In less than a week the man was dead. Autopsy revealed carcinoma of the sigmoid flexure. His reasons for opposing resection of the sigmoid and making anastomosis by the Murphy button were, in substance, the same as Dr. Reed's. Cancer in the sigmoid flexure was not only usually attended by systemic infection, but there is an involvement of other organs and tissues of the body. He would therefore ask, could a man live any longer after a surgeon had removed the tumor than he would if it was left untouched? Granting that there is total obstruction, would it not be better to perform colotomy and let the man live out his allotted days with cancer in a more pleasant way than he would if an operation were done? In lieu of this, it had occurred to him that the plan suggested by Dr. Bacon, of Chicago, of anastomosing the colon to the rectum, leaving the growth there, would be a more favorable operation than ex tirpation of the carcinoma.

Dr. A. M. CARTLEDGE, of Louisville, said in doing a cholecystotomy there was not much time saved by using the Murphy button, and it was not as useful as the ordinary method of suturing. He thought this was well illustrated in one of the cases reported by Dr. Vander Veer in which there was a passage of stones after the operation, and where it was necessary on account of the extremely feeble condition of the patient. In cases with numerous small calculi extending into the cystic and common ducts, he had made a comparatively large incision in the gall bladder and sutured it to the peritoneum, where the stones could not be removed, and they would then pass for days externally through the drainage tube. The orifice in the button is too small to permit the stones to pass, whereas, they would escape through a drain and come out. He expressed himself in favor of Dr. Grant's device, and considered it an excellent one for lateral anastomosis.

DR. W. E. B. DAVIS believes the Murphy button can be used to advantage in intestinal work where it is necessary to do operations quickly; otherwise, the method of stitching similar to that practiced by Abbe is better, is more certain, and accidents are not so likely to follow it as by the use of a mechanical appliance which is non-absorbable. Cholecystenterostomy by the button should be resorted to only in those cases where it is impossible to remove the obstruction in the common duct. The old method, as pointed out by Dr. Cartledge, is decidedly better in the other class of operations.

DR. VANDER VEER, in closing, was satisfied that end-to-end anastomosis with the button in the large intestine was not likely to be a satisfactory procedure on acount of reasons mentioned by Dr. Reed. He believed Dr. Grant had presented an appliance that would be of value to the profession. The fact that new devices were being presented from time to time before medical gatherings for intestinal anastomosis was ample evidence that we had not yet reached an ideal method. The Murphy button is an excellent device for the performance of cholecystenterostomy and other operations.

DR. GRANT believes that any surgeon of ordinary skill with his device, after having the two surfaces of the bowel directly opposed, can suture them without soiling the peritoneum or letting them slip away.

DR. J. MCFADDEN GASTON, of Atlanta, Ga., read a paper on

### SURGICAL INTERFERENCE IN RECTAL DISORDERS.

After outlining the anatomy of the rectum, the author said it is a mooted point in regard to the practicability of eradicating rectal troubles of syphilitic origin by medication, and with the present light on the subject it seems justifiable to resort to such a surgical measure as the condition indicates, while constitutional treatment is being carried out in the case.

There are instances of supposed development of specific disease in the form of stricture of the rectum, after the lapse of many years subsequent to any syphilitic contamination, and some authors claim their ability to diagnosticate specific stricture, even without a previous history of primary syphilis. Strictures of the rectum from fibrinous depositions in its walls call for division or excision of the structures involved. When carcinomatous induration of the rectal tissues is detected early, there is encouragement to undertake an operation, but after the breaking down of the neoplasm with infiltration of surrounding structures, no benefit is derived from excision of the parts involved. The rectum affords material for surgical work of the most important character, and it should not be relegated to those professing to deal with so-called "orificial" surgery. Dr. Gaston is fully impressed with the conviction that many cases find their way into the hands of quacks which ought to be treated by members of the regular medical profession, and preferably by those who have made a special study of rectal diseases and are prepared to treat properly all the surgical disorders of the rectum. Reference was then made to a paper by Dr. Gerster, read before the American Surgical Association, upon the surgery of the rectum, in 1893.

The burning and urgent appeal to the surgeon to-day is for a definite settlement of the issue as to active interference in cases of pronounced cancer of the rectum. Shall we content ourselves with the mere palliative measure of inguinal colotomy and leave the diseased structures untouched, as urged by Dr. Mathews in his paper before the American Medical Association, or shall we endeavor to remove all the tissues involved by extirpation, as recommended by Dr. Gerster? The full statistics of results in the hands of skilled operators ought to be collected and a fair analysis made before a final adjustification of the question can be reached. The materials for such a comparison should be obtained form cancer hospitals in this and other countries, as well as from general hospitals receiving and treating this class of patients, and being grouped together, a fair inference may be drawn as to the feasibility of active interference in any case of carcinoma of the rectum.

Dr. J. M. Mathews, said the essayist, incidentally alluded to fissure of the rectum giving rise to reflexes. He was glad he mentioned this simply to emphasize the point, that to have reflexes from the rectum we must have a pathological condition. The so-called orificial surgeons had run wild with reflexes from the normal rectum, and as a consequence, many respectable citizens in his own city had lost healthy rectums. In regard to stricture of the rectum, his observation has been that benign rectal stricture is very seldom met with. There were cases, however, mentioned by authorities, but Dr. Mathews had failed to find them. If he does find it, it is simply an annular constriction of the mucous membrane, which is easily dissipated. It does not require excision. When the surgeon introduces his finger into the rectum and finds stricture there, it betokens one of three serious diseases—syphilis, tuberculosis or cancer, and the patient should not be turned aside with a jesting remark that he has a rectal stricture. It is a serious thing. He maintains that 60 per cent. of the cases of stricture of the rectum arise from syphilis, or are the result of it. He had asked his professional friends to investigate this matter and make known their investigations. The responses he had received were nearly all in the affirmative. He regards stricture of the rectum as more frequent than either cancer or tubercle.

With reference to excision of the rectum for a cancer that has blocked the rectum to the sigmoid flexure, in nearly every instance we have systemic infection. This being the case, can the man be cured by surgical interference? He wished, like Dr. Gaston, we could successfully remove the rectum for cancer, but he doubts it.

DR. H. M. NASH, of Norfolk, Va, had seen a number of cases of ulcer of the rectum cured by dilatation of the sphincters and topical applications. He uses the Sims speculum, placing the patient in the exaggerated Sims' position which gives the operator all the room he wants for manipulation in the rectum.

DR. GASTON, in closing, congratulated Dr. Mathews on his attitude of masterly inactivity in a great many cases of carcinoma of the rectum.

Adjourned.

FIRST DAY.—AFTERNOON SESSION.

DR. GEORGE H. NOBLE, of Atlanta, Ga., read a paper entitled

### ONE HUNDRED AND SIXTY-SIX CASES OF CANCER OF THE PREGNANT UTERUS, OCCURRING SINCE 1886.

The author's attention was directed to this subject by four cases that came under his observation, and his success in dealing with them had encouraged him to look more carefully into the treatment, and, as a result, he had collected a hundred and sixty-six cases of cancer of the pregnant uterus which had occurred since 1886, the time of the Bar thesis. Dr. Noble then confined himself mainly to the statistics of the treatment and results, referring to Bar, Cohnstein and others for information concerning the age, the period of recurrence, the period of abortions, etc. There were twelve partial amputations of the cervix in the seven months of pregnancy, averaging five and a half months; 96.6 per cent. of the mothers recovered from the operations, while 8.3 per cent. died; 66.6 per cent. went to full term, one child dying subsequently, and 41.6 per cent. aborted. Two mothers had subsequent operations performed for the removal of cancer, but there was recurrence in both cases. Another conceived a second time, and died thirteen days after confinement, of peritonitis. Of the three cases of intravaginal amputation of the cervix, two recovered from the operation, giving a mortality of 33.3 per cent.; the children the same. One mother died of peritonitis, one died suddenly six weeks after confinement, and the third had two subsequent operations for the removal of the malignancy, making an ultimate mortality of 66.6 per cent., and possibly 100 per cent. The intravaginal amputations give a combined mortality from operations, of mothers, 19.3 per cent., of infants, 40 per cent. Sixteen supravaginal hysterectomies were done prior to the seventh month, with a mortality of 6.2 per cent.; six had recurrences of the disease, three had no return, and seven were not observed. There was, therefore, an ultimate mortality of 66.6 per cent. In the nine cases in which the records are complete, thirteen cases were lost, a mortality of 82.5 per cent. Of the remaining three, one went to full term, and the other two were not mentioned. One case aborted thirty-five days after conception, aborted again in forty days, conceived a third time, was delivered normally, and was well five years afterwards. There were 23 vaginal hysterectomies. In two cases, the results were not recorded, leaving 21 cases, all successful. There were seven cases of vaginal hysterectomy in the puerperal period from fourteen to twenty days after abortion or delivery; all recovered.

The total number of abdominal hysterectomies was 16; 12 of these were Freund's operation, one after Mackenrodt's method, and the remainder not described. Of 11 cases, 7 died from the operation, making a death rate of 43.7 per cent. One case had enchondroma of the pelvis, another had return of the cancer in one year, and a third had a return in a few months, and died seven days after an operation for ileus due to cancer of the intestines. These three are the only ones with complete records; therefore, it is impossible to give an estimate of the ultimate recoveries. The products of conception were all lost.

Cæsarean section was done forty-three times as follows: Conservative (or Sanger), 26; Porro, 9; Freund's, 8 times. Of the 26 conservative operations, 16 died, and seven recovered; in two the results are not recorded, and one was dead before the operation was performed, mortality in 23 cases being 43.7 per cent.

The number of recoveries in the Cæsarean-Porro operations was four, deaths five, mortality of 55.5 per cent. In eight Cæsarean sections by the Freund method, there were three recoveries and five deaths, giving a mortality of 62.5 per cent.

A short summary shows: (1) That vaginal hysterectomy should be safe in the early months of pregnancy and the puerperal state, when there is a reasonable hope for the mother. (2) That abdominal hysterectomy should be done when the uterus is too large to be rapidly and safely removed through the vagina. (3) That at or near the end of pregnancy, Cæsarean section should be resorted to when the child's interest is to be considered. (4) That Cæsarean section with Freund's operation is permissible, when the disease is confined to the uterus and the child is viable. (5) That in doubtful cases, cutting of the cervix and rapid delivery may be judicious when the incision can be made in non-ulcerated or non-infiltrated tissue. (6) That as there are four chances to one against the life of the fœtus, and as an equal number of mothers may be ultimately cured in early stages of the disease, the safety of the fœtus should not be allowed to hazard the life of the mother; and that upon the other hand, the futile efforts directed to the interest of the mother, when her case is hopeless, should not jeopardize the safety of the fœtus in the latter months of pregnancy.

DR. HOWARD A. KELLY, of Baltimore, said that cancer of the pregnant uterus is rare. He had seen but three cases. If the cancer is seen in the early stage, when it presents itself as a mere nodule on the cervix, not apparently extending into the broad ligaments and pregnancy is approaching, it would be safe to let pregnancy go on to full term and labor to take place naturally. Per contra, if the cancer has advanced to such an extent that there is a possibility of involvement of the broad ligaments, the surgeon could not operate too soon, because, under the conditions of pregnancy, the growth of cancer of the cervix is much more rapid than it is ordinarily.

DR. VANDER VEER, of Albany, stated that four years ago he operated on a case, doing a vaginal hysterectomy. Pregnancy had advanced about four months. He felt happy about the case for two and a half years, at the end of which time cancerous nodules presented themselves at the site of the cicatrix. Pregnancy had advanced to full term. The patient lived for a period of eight months. The pelvis was filled with a cancerous deposit. Dr. Vander Veer reported another case of cancer of the pregnant uterus, and while the patient recovered from the removal of the uterus, she subsequently died from a recurrence of the disease.

DR. E. S. LEWIS, of New Orleans, contributed a paper entitled

### HYSTERECTOMY FOR FIBROIDS.

The author said that hysterectomy for fibroids, now a justifiable and recognized operation for the preservation of health, the prolongation and saving of life through important operative procedures minimizing the element of risk, had reached its present enviable position by the substitution of direct ligation of the uterine arteries for the unsatisfactory methods heretofore employed to secure immunity from hæmorrhage, these measures often failing to prevent bleeding and not infrequently there was exposure to infection through the region of the cervix. Complete hysterectomy, whether by the vaginal route in fibroids of moderate size, through the abdomen in certain cases, or by the combined abdominovaginal method in other instances, heralded a brighter era for the future of hysterectomy. That exceptions might occur with regard to individual cases, rendering the complete operation inadmissible, he was prepared to admit.

The author then reported eight cases in which the complete operation was practiced.

CASE I.—Patient aged 53 years. Tumor the size of a seven-months' pregnancy. Operation performed March 11, 1894. Incision from pubes to two inches above umbilicus. Omentum detached from anterior surface of tumor, to which it was adherent; a portion of omentum ligatured and cut off on account of free oozing. The subperitoneal fibroid attached to the fundus was lifted out and upper portion of ligaments ligated and divided. The bladder was then detached and the vaginal roof opened in front and behind. The lateral vaginal connections and lower portion of broad ligaments were transfixed and ligated with a double ligature, and the uterus freed. One suture through middle of vaginal vault. The incision (abdominal) closed and the vagina loosely packed with iodoform gauze pushed above vaginal roof for drainage on account of some slight venous oozing. The tumor weighed twenty-five pounds. This patient made a satisfactory recovery.

CASE II.—Patient 43 years of age. Had profuse uterine hæmorrhages. Uterus as large as a five-months' pregnancy from interstitial fibroid. Endometrium curetted and iodine injected without relief. Hysterectomy of the vaginal method. Recovery.

CASE III.—Patient aged 63 years. Diagnosis, interstitial fibroid with carcinoma of the cervix. The uterus reached the middle of the hypogastrium and was about the size of the preceding case. Its removal was easily effected in the same manner. Ligaments were secured with clamps. The fibroid, about the size of an orange, was not weighed. Recovery.

CASE IV.—Patient aged 44. Uterus enlarged from small fibroids. Vaginal hysterectomy, followed by recovery.

CASE V.—Patient aged 35 years. Diagnosis, uterine fibroid, reaching to umbilicus. Recovery.

CASE VI.—Patient aged 42. Large uterine fibroid the size of an eight-months' pregnancy. Vaginal hysterectomy.

CASE VII.—Patient aged 40. Diagnosis of subperitoneal fibroid, attached to fundus. Same operation.

CASE VIII.—Colored woman. Fibroid affecting the body of the uterus and as large as a six-months' pregnancy.

DR. HOWARD A. KELLY, of Baltimore, made some remarks on the

### TECHNIQUE OF SUPRAVAGINAL HYSTERECTOMY.

He described a new method for hysterectomy in removing the uterus, ovaries and tubes through the abdomen. It is a modification of the Baer method. He had tested it in about one hundred and fifty-five cases of all kinds, and had operated in the presence of hundreds of practitioners. While he had not heretofore described it, he had briefly referred to it before the Section on Obstetrics and Diseases of Women at the Baltimore meeting of the American Medical Association.

DR. JOSEPH TABER JOHNSON, of Washington, D. C., followed with a paper entitled

### SEVENTEEN CASES OF HYSTERECTOMY.

The first successful hysterectomy ever performed was done by Dr. Burnham, of Lowell, Mass., in 1853. Kimball, who assisted Burnham in his first case, subsequently operated with success after a correct diagnosis had been made. In 1875, Kimball reported nine hysterectomies, with three deaths. Burnham had then done sixteen hysterectomies, with four deaths. These results were considered fairly good at that time. In 1878, Gusserow reported that up to 1866, Koeberle had lost all but eight out of forty-two hysterectomies, giving him a mortality of 81 per cent. Schroeder collected reports of 108 hysterectomies, with a mortality of 85.3 per cent. Thomas' "Diseases of Women" reports 24 cases, with eighteen deaths. Storer, in 1874, reports ten American hysterectomies; all died. From 1874 to 1894, m any changes in technique, including asepsis, the Trendelenburg position, the intrapelvic but the extraperitoneal treatment of the pedicle, the closure by suture of the separated edges of the broad ligament, drainage when neccessary through the vagina after total extirpation, have all had their share in diminishing the mortality from 85.3 per cent.

In the June number of the *Annals of Gynæcology*, Cushing published a report of 1,670 suprapubic hysterectomies done by American operators, with a mortality reduced to 13.8 per cent. One of the improved methods of widening the scope of this beneficent and magnificent operation and greatly reducing its mortality was introduced, advocated and practiced by Dr. B. F. Baer, of Philadelphia, who is quoted in Cushing's article as having operated seventy-eight times, with seventy-one recoveries and seven deaths.

Dr. Johnson presented his paper for the purpose of reporting seventeen operations by Baer's method with sixteen recoveries and one death.

The three preceding papers were then discussed jointly.

DR. HENRY O. MARCY, of Boston, agreed with Dr. Johnson that the method of operating by the Koeberle clamp had been shown to be absolutely wanting in this type of operation; that it is not easier to do excepting in very few and rare cases; that it is ill-advised and subject to serious dangers.

In reference to the operations described by Dr. Kelly, he desired to refresh the memory of the members that in 1880, he published a paper in which he reviewed the various steps and pointed out the advantages of the operation which is now known under the name of Dr. Baer. In the International Congress of 1881, he presented a second paper in which he emphasized the value of it. The advantages of leaving the stump was a great gain in subsequent result, in that it left a sort of fixation point between the uterus, rectum and bladder. It is of value, again, in that it does not shorten the vagina—a question of paramount importance in reference to marital life, and of the conditions that may follow in the subsequent history of the patient. He was sure that surgeons were working on the line of great and general improvement, and that when the technique of this operation has been developed more thoroughly, the time is coming when the removal of large fibroid tumors of the uterus will be accomplished with equal safety as the removal of large ovarian cystomata.

DR. RICHARD DOUGLAS, of Nashville, said one advantage of the procedure described by Dr. Kelly was that the bladder was out of the operative field, the surgeon not having to handle that viscus; that the operator could open the broad ligament and remove the interligamentary fibroids without difficulty and without danger. Another important feature was that the risk of ligating the ureter was greatly reduced.

DR. W. E. B. DAVIS, of Birmingham, believes that most surgeons are now inclined to accept the intra-abdominal method of operating, although it was still a question whether we shall have a pedicle or go in through the vagina. A very important point was with reference to the time of these operations. He had seen Dr. Kelly operate and admired his manual dexterity; but to take out the uterus in six or seven minutes meant very little, when it takes an assistant, or the operator himself, an hour or more to complete the balance of the operation. At the meeting of the American Medical Association, he saw Dr. Kelly remove the uterus by the method he had described, in something like seven minutes, but it took his (Kelly's) assistant one hour and twenty minutes to finish the operation. This was no reflection on Dr. Kelly's skill as an operator, but he thought the matter of time should go on record in a little different way.

DR. A. VANDER VEER, of Albany, remarked that in 1889, he removed the uterus somewhat in the manner described, the case having been already recorded. He had done the operation only twice since. He uses the Koeberle clamp because he thinks he can do the operation much quicker, and has no reason to regret its use.

DR. A. M. CARTLEDGE, of Louisville, believes it is an advance in pelvic surgery to remove the uterus in badly septic cases, whereas, formerly, only the tubes and ovaries were taken out.

DR. E. S. LEWIS, of New Orleans, remarked that his experience with total hysterectomy for fibroids was restricted to the cases he had reported. The operations which he had performed up to last year were cases in which a partial removal of the uterus was effected, leaving a portion of the cervix. He could not but think, however, notwithstanding the majority of the gentlemen who had spoken upon the subject appeared to be advocates of the partial operation of leaving a part of the cervix, that total removal of the uterus was the best operation.

DR. KELLY said, in reply to Dr. Davis, that when he spoke of removing the uterus in seven minutes or less, it did not include closure of the wound, dressing, etc. He had never been so dishonest as to make the statement that the entire operation could be done in such a short time, nor would he like that impression made.

DR. JOHNSON remarked, in reference to taking out the cervix, that it prolongs the operation and necessitates greater mutilation and more stitching, and seems to be unnecessary in view of the successful cases that had been and are being reported. Patients get perfectly well without doing it.

He concurred in Dr. Davis' statement that the time consumed in taking out the uterus did not amount to much, but that we should consider the matter from the time the first incision is made until the last stitch is inserted, the wound dressed, and the patient off the table.

DR. JOSEPH PRICE, of Philadelphia, contributed a paper entitled

### ABDOMINAL HYSTERECTOMY.

Among other things, Dr. Price said that had the same mortality attended the early ovariotomies that attended the first ventures in hysterectomy, there would have elapsed a longer period than forty or fifty years between the first successful ovariotomies and the date of the revival of the procedure. Both vaginal and supravaginal hysterectomy had been largely practiced by those who had given pelvic surgery most attention. They are the men who contributed the most to perfecting the procedure. Abdominal hysterectomy is the one procedure indicated in all cases of intrauterine malignancy, where vaginal portions of the cervix are not involved, in all cases of uterine malignancy complicated with tubal and ovarian diseases, and in cases of uterine fixation antedating the malignant development. Hysterectomy, vaginal or supravaginal, should be a simple, direct and complete operation in every detail. Where the operation is done with good surgical skill and judgment, there will be comparative immunity from all risk of dangerous hæmorrhage, and avoidance of sepsis. The method of procedure successful experience recommends as safe, the most satisfactory and complete in its results, is extirpation by lateral ligation, incision of the posterior vaginal fornix, circular incisions of vagina to bladder; and approximating vaginal walls to and matching perineum, completes the simple procedure. This is the method with which others, as well as Dr. Price, have met with the best success. Operations for malignancy by the proper method of dealing with omental and other adhesions lessen the risk of post-operative troubles and early recurrence. In all operations for malignancy, Dr. Price advises the removal of both the ovaries and tubes, and says that an operation would be imperfect without it. The leaving of the ovaries sometimes results in the growth of small tumors, necessitating an additional operation. The toilet should be perfect. If the operation has been complicated by pus accumulations in tubes and ovaries, with universal adhesions, irrigation, followed by glass drainage, will give the best results. Drainage should be used in all cases where the adhesions are extensive, as oozing of blood and serum may be very free.

Morcellation, with mortality of one in seven, cannot certainly be said to offer any very strong claim to our consideration. Such a mortality does not compare favorably with the much abused extraperitoneal operation with Koeberle, or elastic ligature. Dr. Price had no statistical reasons for complaining of any of the four methods—intraperitoneal amputation, extirpation, supravaginal, extraperitoneal, or vaginal extirpation, as his results in all had been altogether satisfactory from the standpoint of recoveries.

### HYSTERECTOMY IN ACUTE PUERPERAL SEPSIS, WITH REPORT OF CASES.

DR. A. M. CARTLEDGE, of Louisville, Ky., reported two very instructive cases, after which he summarized his conclusions with reference to acute puerperal sepsis as follows:

1. From our present knowledge of the causation and nature of puerperal infection, we may say it is largely a preventable disease.

2. When occurring, it is of the greatest importance to differentiate between puerperal intoxication or invasion of a piece of putrescent placenta or blood clot by saprophytic germs, and true septic infection or invasion of living cells by pathogenic bacteria. Puerperal sapræmia, though in many cases producing the most alarming symptoms, is usually amenable to energetic treatment by curettage, antiseptic irrigation and satisfactory tubular drainage of the uterine cavity.

3. True septic infection should be treated by sterilizing the birth canal at the earliest possible time, free elimination by purgation and a prompt evacuation of superficial abscess accumulations about the cervix. Such a course may save the patient from more radical measures.

4. The chief differential points between puerperal intoxication and true puerperal infection are the comparative absence of pain, tympanites and abdominal tenderness, and the more sudden onset and severe character of the symptoms in puerperal intoxication. Hysterectomy, as a primary measure, is never justifiable in septic intoxication, and when necessary, it can only be after the mixed or secondary infection which may follow in the track of a primary sapræmia.

5. Progressive involvement of the deeper structures, as evidenced by daily elevation of temperature, probably 103 degrees F. in the evening and subnormal in the morning, to gether with night sweats, scanty secretions, ascending pulse, are indications for hysterectomy.

6. It is often impossible, from the involvement primarily of the low pelvic structures, to make a bimanual examination which will reveal the true condition of the uterine appendages. But in view of the fact that these structures are not so prone to be invaded in the acute violent type of the disease, vaginal hysterectomy should be the operation of selection.

SECOND DAY.—MORNING SESSION.

DR. RICHARD DOUGLAS, of Nashville, read a paper entitled

### SPLENECTOMY STATISTICALLY CONSIDERED, WITH REPORT OF A CASE.

Gathered from all sources, the author finds on record 194 splenectomies. Of these, 126 were females, 57 males and in 11 cases the sex is not given. Furthermore, he finds that in 40 cases the operation was undertaken for wounds or injuries. Of this number, 26 were males and 14 females. If we deduct these, we find that the ratio of splenectomies for disease is 31 males to 112 females, showing the latter sex to be much more predisposed to disease of this organ.

Dr. Douglas then reported the following case: Mrs. J. S., aged 33, housewife, multipara, native of Tennessee, family history good, has suffered occasionally with menorrhagia, but more recently from amenorrhœa. She had malarial fever when twelve years of age. The last three years she has lived in the western portion of the state, on the banks of the Mississippi, and has suffered during this time from frequent attacks of intermittent malaria. About August 1, last, she suffered an acute pain in the left side. A tumor was then discovered in that region about the size of a fist. Physical examination revealed a smooth, elastic, movable tumor, filling the left lateral region of the abdomen, its borders well defined, edges sharp and notched. It frequently changed its form; at times it appeared flat and smooth, again it would rise up as a sharp ridge extending from ribs to symphysis pubis. There was absolute dullness over the tumor. Vaginal examination revealed the uterus forward, the pelvis filled with a smooth, hard mass, which, upon change of posture, disappeared from the pelvic and occupied the left iliac fossa. She suffered with paroxysmal pain, although not severe. A sense of weight, a dragging in the left side, flatulency, nausea, and, occasionally, vomiting. There was some emaciation and slight anæmia. There was no œdema, ascites, vertigo or insomnia. A blood examination of the case constituted a part of the report.

A diagnosis of malignant hypertrophy of the spleen was made, and after due preparation, the abdomen was opened by a lateral incision at the outer border of the left rectus, the incision being about six inches long. The spleen was found displaced and free from adhesions. Its pedicle was secured by interlocking ligatures; pedicle was severed close to the organ. As additional security against hæmorrhage, ligatures *en masse* were employed; also individual deligation of splenic artery. After removal of the spleen, bleeding from abdominal incision became very profuse and required several ligatures. Peritoneum closed by separate silk sutures, and the abdominal wall coapted by usual interrupted silkworm gut sutures.

The post-operative history of this case was a very stormy one, but one month after operation she was out of bed and is now well.

DR. W. E. B. DAVIS, of Birmingham, contributed a paper entitled

### SURGERY OF THE BILIARY DUCTS.

He reviewed the operative procedures practiced on the biliary passages, and recommended for cases of obstruction from stone in the common and hepatic ducts, that the obstruction should be removed, and that no attempt be made to suture the incision in the duct or ducts. His experiments had demonstrated that the field of operation will be walled off, and that no general inflammation will occur after this treatment. He had tested the value of gauze in draining bile in injuries of the gall bladder and ducts, and reported cases where he had removed the gall bladder without tying the duct, by packing with iodoform gauze. The animals got well. In other instances where he incised the gall bladder and ducts and packed with gauze around the openings, no stitches being used, the animals recovered. Complete walling off of the general cavity was noted when the abdomen of the animals was reopened. The experiments of Dr. Davis demonstrated conclusively that the peritoneum is capable of taking care of a small amount of bile, but the large quantities or the constant extravasation of it will produce a fatal peritonitis, usually in from twenty-four to forty-eight hours.

### MANAGEMENT OF CASES WHICH HAVE RECOVERED FROM APPENDICEAL ABSCESSES IN WHICH THE APPENDIX WAS NOT REMOVED.

DR. JOHN D. S. DAVIS, of Birmingham, Ala., read this paper. The practice of dealing with appendiceal abscess by simply evacuating the pus and draining the cavity thoroughly without any very extensive search, or the breaking up of adhesions in order to find the appendix, has been adopted by a large number of the leading operators for some time. More recently, some of the leading surgeons have advocated in all cases that the operation should be made complete, that all adhesions should be freed and the appendix removed. One leading abdominal surgeon, who has, perhaps, done more work in pelvic surgery than any other man in this country, has advocated this plan of treatment in vigorous terms. In a large proportion of cases of pus in the tubes and ovaries gonorrhœa has been an important factor in its production. Such pus is not septic and is not calculated to give rise to so dangerous a general inflammation as infection from an appendicitis or an appendiceal abscess. It is a notable fact that a ruptured tube or ovary will usually be followed by a circumscribed inflammation. It is the exception that a fatal general peritonitis results from such an accident. The most fatal forms of peritonitis are due to a ruptured appendiceal abscess. In fact, but few cases are saved when such an abscess ruptures into the general cavity.

An operation on an appendiceal abscess is usually one of the simplest procedures and is attended with almost no danger. Where the inflammation is circumscribed and the drainage is thorough nearly all cases recover. The records of operations for appendiceal abscesses show that the great majority of cases are cured after evacuation and complete drainage. Recurrence of the disease in such cases is rare. The appendix, in large proportion of cases having ruptured before the abscess formation, is completely drained through the abscess and permanently cured. In others the appendix is destroyed by the inflammation and there is nothing left of it when the abscess is operated upon. To make an extensive search for the appendix is liable to break up adhesion and then allow escape of septic fluid into the general cavity. Thus a very simple condition may be converted into one of the most serious that could happen to the peritoneal cavity. Dr. Davis believes that there cannot be much need of breaking up adhesions, for they give way in a short time after the abscess is relieved. In breaking up these adhesions, in addition to the danger mentioned, the surgeon prepares a favorable condition for fresh adhesions, with the possibility of the bowel being fastened in a position that will produce pain and often obstruction. After the abscess is thoroughly cleaned out, gauze packed into the abscess cavity and between the abscess and abdominal wall, will completely shut it off, and the chances for recovery will be good in such cases. Dr. Davis does not favor the breaking up of adhesions and searching for the appendix in cases of appendiceal abscess.

SECOND DAY.—AFTERNOON SESSION.

DR. JOHN A. WYETH, of New York City, delivered a memorial address on

### DR. J. MARION SIMS AND HIS WORK.

Dr. Wyeth said it was safe to say that Marion Sims attained the highest position ever achieved in the history of the profession. He stands alone in this: his reputation as a surgeon was so world-wide that in any capital, in any country within the domain of civilization, he could command at any time a lucrative practice. Assuredly, there does not exist in the history of surgery another such distinction. In New York, London, Paris, Brussels, Berlin, Vienna, Rome, Madrid, Lisbon and St. Petersburg, he found himself everywhere sought after, not only by the patients he could benefit, but by the leading members of his own profession, who were anxious to pay tribute to his wonderful genius. The study of his life should instill hope into the heart of every student. Born amid the most humble conditions in a backwoods county of South Carolina, he died the foremost surgeon of his country and of the world. What a transition! From the log cabin of a poor farmer in Lancaster District, to the palace of Saint Cloud, where he was the guest of Napoleon III., the trusted physician to the empress, as he was to the highest and lowest of those who sought relief at his hands in any part of Europe!

Toward the higher and purer civilization, the progress of man is slow. As yet, the shadows of barbarism linger about him. His heroes are the destroyers, the Cæsars and Napoleons, who covered the earth and buried beneath it lives, sacrificed upon the altar of personal ambition. But the time must come when those whose genius and works give life and health and happiness to the world will be the first in the heart of man. In this purer temple of fame, along with those of Jenner, Ephraim McDowell, Morton, Lister, Pasteur and others, generations yet unborn shall read the name of Marion Sims.

At the close of Dr. Wyeth's address, remarks were made by Drs. Robinson, Wilson, Nelson, Marcy, Engelmann, Kollock, Vander Veer, Gaston, Tiffany and Westmoreland, eulogizing Sims, most of whom had enjoyed his personal acquaintance.

DR. GEORGE BEN JOHNSTON, of Richmond, Virginia, read a paper entitled

### COMPARATIVE FREQUENCY OF STONE IN THE BLADDER IN THE WHITE AND NEGRO RACES.

It is commonly stated by writers on urinary diseases that stone in the bladder is a rare occurrence in the negro race. This is so at variance with the author's own experience, that he has instituted an investigation either to prove the statement, or to correct the fallacy. He selected the southern states of Virginia, North Carolina, South Carolina, Alabama, Georgia, Tennessee, Kentucky, Florida, Louisiana, Mississippi, Arkansas and Texas as the field of inquiry. He selected four hundred representative practitioners to correspond with in order to procure the necessary data. He received 338 responses, 94 of which contained information, and the remainder were negative. He succeeded in collecting 1,068 cases of stone in the bladder. Of these, 952 were in white subjects and 116 in negroes. It is at once observed that the negro cases represent 9.55 per cent. of all cases reported. This showing is quite sufficient to disprove the idea of immunity, which the negro is supposed to enjoy.

Geographically these stones were distributed as follows:

| | |
|---|---|
| Alabama | 10 |
| Arkansas | 11 |
| Florida | 28 |
| Georgia | 90 |
| Kentucky | 56 |
| Louisiana | 19 |
| Mississippi | 9 |
| North Carolina | 126 |
| South Carolina | 66 |
| Tennessee | 128 |
| Texas | 98 |
| Virginia | 447 |

Sex is specified in 780 cases, and not stated in 280. Of those in which the sex is indicated, there were 691 in males, and 97 in females, or about seven times oftener in males than in females.

There were 182 cases not subjected to operation and 584 in which the stones were removed by the following methods: Lateral perineal, 249; median perineal, 100; suprapubic, 138; vaginal incision, 32; dilatation of female urethra, 28; crushing, 35; operation not given, 5.

Of those operated on, 541 recovered and 43 died. No report of operation in 304 cases. Dr. Johnston's own cases are incorporated in the foregoing statistics. During his twenty years practice he has made notes in 41 cases. Of these, there were 35 in whites, 6 in blacks, 39 in males, and 2 in females. Thirty-nine were operated on and 2 were refused operation on account of advanced kidney disease. Both died. In 25 cases, lateral perineal lithotomy was done; in 12, suprapubic; and in the cases of the two females, the urethra was dilated and fragmentation practiced. He had no deaths following his operations.

### PRESIDENT'S ADDRESS.

This was delivered by Dr. L. McLane Tiffany, of Baltimore, Maryland.

He said the aim of the Association was twofold; namely, first, to do advanced work, and second, to bring it to the notice of, and aid other members of the profession. The fellowship of the Association was a very extended one, embracing a large territory, with infinite varieties of soil, temperature, environment, etc., yet the transactions did not show those local records from which facts may be generalized, applicable to the area from which the fellowship is drawn. It did not seem reasonable to him to suppose that a surgical operation done among the mountains of western North Carolina was going to behave quite the same way that a similar operation would if done on the Gulf coast of Texas. It did not seem reasonable that similar surgical operations on the banks of the Mississippi and the central plateau of Tennessee would behave the same way. Accurately kept charts with exact and careful notes would unquestionably show differences not yet placed on record by anyone, from which much clinical information could be learned. Again he questioned whether surgical operations undertaken during the great heat of summer, or after the long continuance of summer heat, would show similar charts or give like results when compared with operations upon patients not subjected to high atmospheric temperature, either temporary or long continued. No association had a membership better situated or more competent to carry on a series of such investigations.

DR. WILLIS F. WESTMORELAND, of Atlanta, made some remarks on

### CYSTOTOMY FOR STONE.

The author said that any surgeon of the present day who had had a long and extensive experience in operating for stone, must acknowledge that the upper operation is better and safer. Unless there is a pathological condition of the blood or infection of the bladder, as recognized by chemical or microscropic examination, the surgeon could decide before operation what course to pursue. Dr. Westmoreland said that the anatomy of this region, as laid down by the investigations of Strong and Petersen upon the cadaver and frozen sections, led the surgeon astray, and the observation of the surgeon is more to be depended upon than any literature we have thus far pertaining to the subject. In operations for stone, the author said he does not care whether he sees the base of the bladder or not; that he depends upon touch, and that therefore rectal distension might be dispensed with. In the place of rectal distension, he recommends that a vessel of water be suspended three or more feet above the patient, according to the amount of distension necessary. Where a surgeon desires to effect distension of the bladder by a vessel, if the bladder is ulcerated at any point, with a thickening here or thinness there, it could be done without sudden force, and if the patient during an operation should sneeze or cough, or contraction of the bladder take place, instead of contracting upon a solid mass of fluid, the fluid is forced back into the vessel and there is practically no increase in pressure.

DR. CORNELIUS KOLLOCK, of Cheraw, S. C., read a paper on

### ABDOMINAL PREGNANCY.

After referring to the pathology of extra-uterine fœtation and the classification of its varieties by early writers, he reported the following interesting case: October 18, 1894, he saw for the first time a dark mulatto, 34 years of age, the mother of three children, whose general health had been good until within the last fifteen months. The abdomen was very much distended, measuring at the umbilicus 63 inches. Fluctuation was evident and wave tap very distinct. The patient affirmed that she was pregnant, and that she had gone four months beyond the actual period of gestation. After a thorough examination, laparotomy was decided upon, and an incision was made four inches in length below the umbilicus. The walls were so thin that the instrument penetrated the cavity before it was certain that the abdominal muscles were divided. There was a sudden and copious discharge of offensive matter. An immense fibroid was removed from the anterior portion of the sac. The cavity also contained a fœtus weighing ten pounds. The placenta had undergone fibroid degeneration, with only a small part of the placental tissue remaining. The patient was extremely weak when operated on. She lived for five or six weeks after operation, and Dr. Kollock thinks she would be alive to-day were it not for the unfortunate intervention of intestinal obstruction.

DR. J. T. HENRY, of Chester, S. C., reported a case of

### EXTRA-UTERINE PREGNANCY.

The abdomen was freely opened and a large dark mass the size of the head of an adult came into view. The uterus was crowded very much forward. The mass lay posterior to it and was very much adherent to the fundus posteriorly and to the promontory of sacrum. The fimbria of the right tube spread out over the covering of the mass. It was with some difficulty that the mass was freed from its attachments except that portion to the fundus of the uterus, and it was thought best to remove the uterus with it, which we did after tying and cutting the broad ligaments. The fœtus was five inches long and lay between the placenta and the uterus, the cord being attached to the left margin of placenta. The abdomen was thoroughly washed out with sterilized water and closed without drainage. Patient sat up on the fourteenth day after operation, was out of bed in twenty-five days and has gained 25 pounds in weight.

### THE TECHNIQUE OF THE BURIED SUTURE.

DR. HENRY O. MARCY, of Boston, Mass: The constant receipt of letters from all parts of the country containing inquiries concerning the method for the safe application of the buried animal suture, prompted the author to write this contribution. At the risk of seeming dogmatism, he would venture to assert that aseptic wounds, with very few exceptions, should be primarily closed by buried tendon sutures and hermetically sealed with iodoform collodion. Carefully selected tendons are to be preferred for buried sutures, since primarily their anatomical construction makes them stronger, more compact, and, as a consequence, more resistant to the softening processes which must ensue when buried in the living structures. When properly preserved, they have not been subject to bacterial decomposition, and hence may be sterilized without detriment to their ultimate elements. When tendon has been chromicized, it is best preserved in a sterilized oily fluid. Experience has shown that by far the best preserving fluid is linseed oil sterilized by heat, to which carbolic acid has been added. Tendon improves so much when thus kept that Dr. Marcy rarely uses it until it has been in carbolic acid from three to six months. A method far too common has been to preserve chromicized catgut and tendon in absolute alcohol, boiled under pressure. There is no question but that such material is absolutely sterile, but the important factor has been singularly overlooked, that by this process the chromic acid is dissolved out of the tendon, thereby leaving it less valuable than if chromic acid had not been used.

The infection of wounds may never be absolutely prevented, but the experience of surgeons teaches us daily to what a marvelous extent it can be minimized, reduced in aseptic wounds to less than five per cent. Not long ago the author examined his own personal experience, reviewing 600 operations with only two per cent. of septic cases—evidence ample to show the safety of the coaptation of wounds by means of the buried animal suture.

One of the interesting features of the meeting was the presentation to the Association of a gavel made from the legs of the operating table used by Dr. J. Marion Sims in his office for twelve years preceding his death. It was the gift of his son, Dr. H. Marion Sims.

The following officers were elected:

President—Dr. E. S. Lewis, New Orleans, La.

First Vice President—Dr. Joseph Taber Johnson, Washington, D. C.

Second Vice President—Dr. Richard Douglas, Nashville, Tenn.

Secretary—Dr. W. E. B. Davis, Birmingham, Ala.

Treasurer—Dr. A. M. Cartledge, Louisville, Ky.

Place of Meeting—Nashville, second Tuesday in November, 1896.

Chairman of Committee of Arrangements—Dr. W. D. Haggard, Nashville, Tenn.

